# Extrapolating Survival from Randomized Trials Using External Data: A Review of Methods

**DOI:** 10.1177/0272989X16639900

**Published:** 2016-07-10

**Authors:** Christopher Jackson, John Stevens, Shijie Ren, Nick Latimer, Laura Bojke, Andrea Manca, Linda Sharples

**Affiliations:** MRC Biostatistics Unit, Cambridge, United Kingdom of Great Britain and Northern Ireland (CJ); University of Sheffield School of Health and Related Research (ScHARR), Sheffield, United Kingdom of Great Britain and Northern Ireland (JS, SR, NL); University of York, Heslington, United Kingdom of Great Britain and Northern Ireland (LB, AM); University of Leeds, Leeds, United Kingdom of Great Britain and Northern Ireland (LS)

**Keywords:** internal medicine, detailed methodology, survival analysis, technology assessment, multiparameter evidence synthesis

## Abstract

This article describes methods used to estimate parameters governing long-term survival, or times to other events, for health economic models. Specifically, the focus is on methods that combine shorter-term individual-level survival data from randomized trials with longer-term external data, thus using the longer-term data to aid extrapolation of the short-term data. This requires assumptions about how trends in survival for each treatment arm will continue after the follow-up period of the trial. Furthermore, using external data requires assumptions about how survival differs between the populations represented by the trial and external data. Study reports from a national health technology assessment program in the United Kingdom were searched, and the findings were combined with “pearl-growing” searches of the academic literature. We categorized the methods that have been used according to the assumptions they made about how the hazards of death vary between the external and internal data and through time, and we discuss the appropriateness of the assumptions in different circumstances. Modeling choices, parameter estimation, and characterization of uncertainty are discussed, and some suggestions for future research priorities in this area are given.

Models for health economic evaluation typically use observed data from randomized controlled trials (RCTs) comparing survival (or times to other events) between competing alternative interventions. However, the choice of intervention will often affect outcomes over a longer period than the follow-up time of the RCTs. Policy makers responsible for making funding decisions will then require estimates of expected survival for a longer period, and a lifetime horizon is often appropriate.^1^ If the observed follow-up time covers a sufficiently large proportion of the overall survival time, then parametric models could be used to extrapolate the observed trends in the hazard of death for each treatment arm. This is the conventional approach to long-term survival estimation in health technology assessments,^2^ but it assumes that the observed hazard trends will continue into the long term, which becomes less plausible as the unobserved period increases. The extent of uncertainty surrounding any extrapolation should also be quantified,^1,3^ and this is difficult to determine from short-term data alone for the same reason.

In general, long-term survival can be reliably estimated only if there are long-term data, since the impact of long-term modeling assumptions on the decision can be substantial.^4^ Since maximum follow-up in clinical trials is typically only 1 to 5 y, some external information is required. This could be taken from a disease registry, cohort or the general population, a formally elicited expert belief, or a combination of observed data and informal assumptions. Most simply, the external “information” could consist of a defensible clinical belief that the risks of death will continue in a particular way in the long term. The National Institute for Health and Care Excellence (NICE) for England and Wales^1^ recommends that any extrapolation should be assessed by “both clinical and biological plausibility of the inferred outcome as well as its coherence with external data sources,” although it does not suggest specific methods to do this. A number of other national funding agencies have a similar requirement for long-term outcomes predictions.^5^ This article discusses methods that have been applied to use external data explicitly to facilitate survival extrapolation, as well as their merits in different circumstances. Below we describe the scope and provide the terminology used throughout the article.

We consider situations where we have both of the following sources of data.

- RCTs providing estimates of the relative treatment effect on survival for the patients of interest, with individual-level survival or censoring times available for at least 1 treatment arm (either directly or estimated from published Kaplan-Meier curves^6^).
- Information on longer-term survival from another source, describing a population with some characteristics (to be discussed later) in common with the patients of interest. After some adjustments, these data can be used to estimate the baseline long-term survival of the patients of interest. If any treatments are given, this is unrecorded, so these data give no information about intervention effects.

We assume the trial data are representative of the population for which the decision is required. In practice, however, given the selection criteria of trials, this will not always be strictly true,^7–9^ which we will briefly discuss at the end of the article.

The data and extrapolation problem are illustrated by the hypothetical survival curves in Figure 1. Each of the 3 “observed” curves are representative samples of survival from the populations labeled A, B, and C. The population of interest receiving a control intervention is labeled A, the population of interest receiving the intervention of interest is labeled B, and the external population is labeled C. The survivor functions assumed to generate each data set are labeled $S_{A}(t)$, $S_{B}(t)$, and $S_{C}(t)$, respectively. We also define the cumulative hazard $H_{k}(t)=-\log(S_{k}(t))$ and hazard (or mortality) $h_{k}(t)=dH_{k}(t)/\mathrm{dt}$ for each group k=A,B,C. The main quantity of interest, the difference in expected survival between interventions, is

$$\int_{0}^{t_{\max}}\left\{S_{B}(t)-S_{A}(t)\right\}\mathrm{dt},$$

which is illustrated by the shaded area between the 2 curves. The upper limit $t_{\max}$ is commonly infinite, giving the lifetime incremental survival.

**Figure 1:**
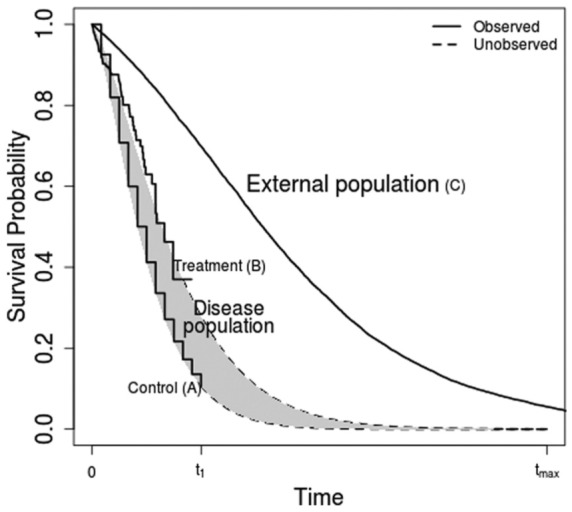
Example survival data. The aim is to extrapolate the incremental expected survival between interventions (B–A) by using long-term data from an external population (C).

In the conventional approach,^2^ $S_{A}(t)$ and $S_{C}(t)$ are estimated by parametric models fitted to the A and B data for $t<t_{1}$, which are extrapolated to $t>t_{1}$ to obtain the incremental survival, without explicitly considering external long-term validity. Instead, we discuss approaches that combine the information on $S_{B}(t)$ and $S_{A}(t)$ for $t<t_{1}$, with external information on $S_{C}(t)$ for $t<t_{\max}$, through assumptions about:

- How survival will differ between the population of interest and the external population. Specifically, how $S_{C}(t)$ compares to $S_{B}(t)$ and $S_{A}(t)$ in the interval $t<t_{1}$ may give information about how $S_{C}(t)$ compares to the disease population survival after $t>t_{1}$.
- How observed survival trends under each intervention will continue in the long term, that is, how $S_{B}(t)$ and $S_{A}(t)$ for $t>t_{1}$ are related to $S_{B}(t)$ and $S_{A}(t)$ for $t<t_{1}$.

Commonly, instead of using this formula directly to calculate the incremental survival, $S_{A}(t)$ and $S_{B}(t)$ are used to obtain parameters in state-transition or similar decision-analytic models, which also allow discounted expected costs and quality-adjusted survival to be estimated for each competing alternative. In this article, we focus on how $S_{A}(t)$ and $S_{B}(t)$ themselves can be estimated using external long-term data and what assumptions are necessary to enable their estimation.

To find methods that have been used for survival extrapolation in cost-effectiveness analysis using external data, we searched the reports of studies carried out under the National Institute of Health Research Health Technology Assessment Programme in the United Kingdom and searched academic literature, focused on health economics and medical statistics journals, using “pearl-growing” search methods.^10^ The exact search strategy, and a broad classification of the 38 relevant papers that we found, are given in the online appendix. In this article, we summarize the methods that have been used, discuss their appropriateness in different circumstances, and suggest where further research might be focused.

## Potential External Data Sources

The long-term survivor function for the external data source $S_{C}(t)$ may be estimated from national administrative data on population survival, disease registries, cohort studies, or elicited expert belief. Typical life-tables published by national statistics authorities provide age, sex, country, year, and cause-specific annual survival probabilities, which can be used to estimate lifetime survival for the general population. External data may also consist of cohorts of patients who are similar to the patients of interest. This could include national or regional registries (such as cancer registries), or hospital-based cohorts including all patients with a particular condition or receiving a particular treatment, from a particular period of time. There may even be data from randomized trials in a similar population with a longer follow-up. The advantages of registry or cohort data compared to unselected national population data are that the patient population may be more representative of the target population, and relevant covariates are more likely to be recorded. However, they may not necessarily have follow-up times covering the whole lifetimes of all participants.

## Framework for Survival Extrapolation Using External Data

**Figure 2:**
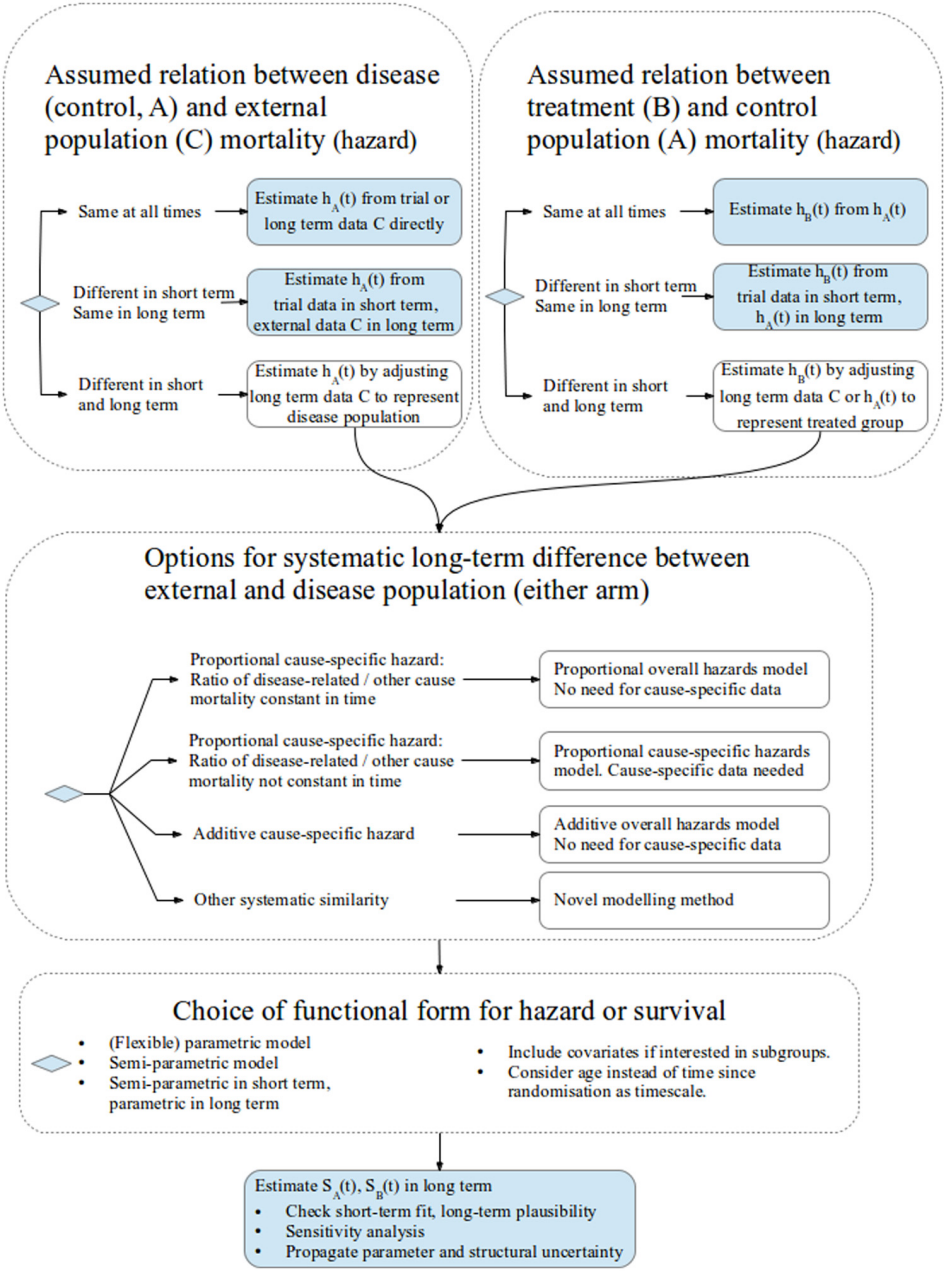
Framework of model choices for survival extrapolation using external data. Long-term survival *S* for control and treatment groups A and B is estimated via assumptions about equivalence of hazards *h* between populations A, B, and C.

Figure 2 illustrates the choices that need to be made when using external data for survival extrapolation. The structure is based on our categorization of different methods used in the literature and our judgment of when they are appropriate. Each of the next few sections of the article discusses a different portion of the figure in detail. Here, we give a brief overview.

First, researchers should identify if the external population (C) has the same mortality at all times, or at least in the long term, as that of the disease population receiving a control intervention (A, top-left panel) and the disease population receiving the intervention of interest (B, top-right panel). In this case, the data can then be used directly to estimate each $S_{k}(t)$ without adjustment.

Otherwise, the long-term mortality of populations A and C (and/or B and C) is assumed to be different but is systematically similar in such a way that the external data (C) can be adjusted to estimate the long-term mortality for the target population with the disease (A or B). The assumptions that have been used to do this are represented by the large middle panel of the figure.

Once any systematic similarity between the internal and external data has been characterized, completing the analysis requires a choice of the functional form for each of the $S_{k}(t)$, potential covariate or subgroup adjustment, parameter estimation, uncertainty, and sensitivity analysis. These issues are discussed later. Some suggestions for future research priorities are made, concentrating on how uncertainty about assumptions is represented and the role of “soft” or elicited information.

## Difference in Mortality Between the Disease and External Populations

### Disease and External Populations Have the Same Mortality at All Times

Sometimes, the disease or baseline intervention of interest is not expected to affect mortality; for example, it may affect only quality of life. Then, long-term survival of the patients of interest can be assumed to be the same as that of the national population of a similar age and sex distribution and taken directly from the relevant life-table.^11,12^

$$S_{A}(t)=S_{C}(t)\;\mathrm{for all t}.$$

This assumption may also hold if the disease or baseline intervention affects mortality, but the external data come from a disease registry or cohort of patients having the same disease and/or intervention, so that the survival of the control group in the trial data is the same as that of the external population.^13–19^

### Disease and External Populations Have the Same Mortality after Some Time

**Figure 3:**
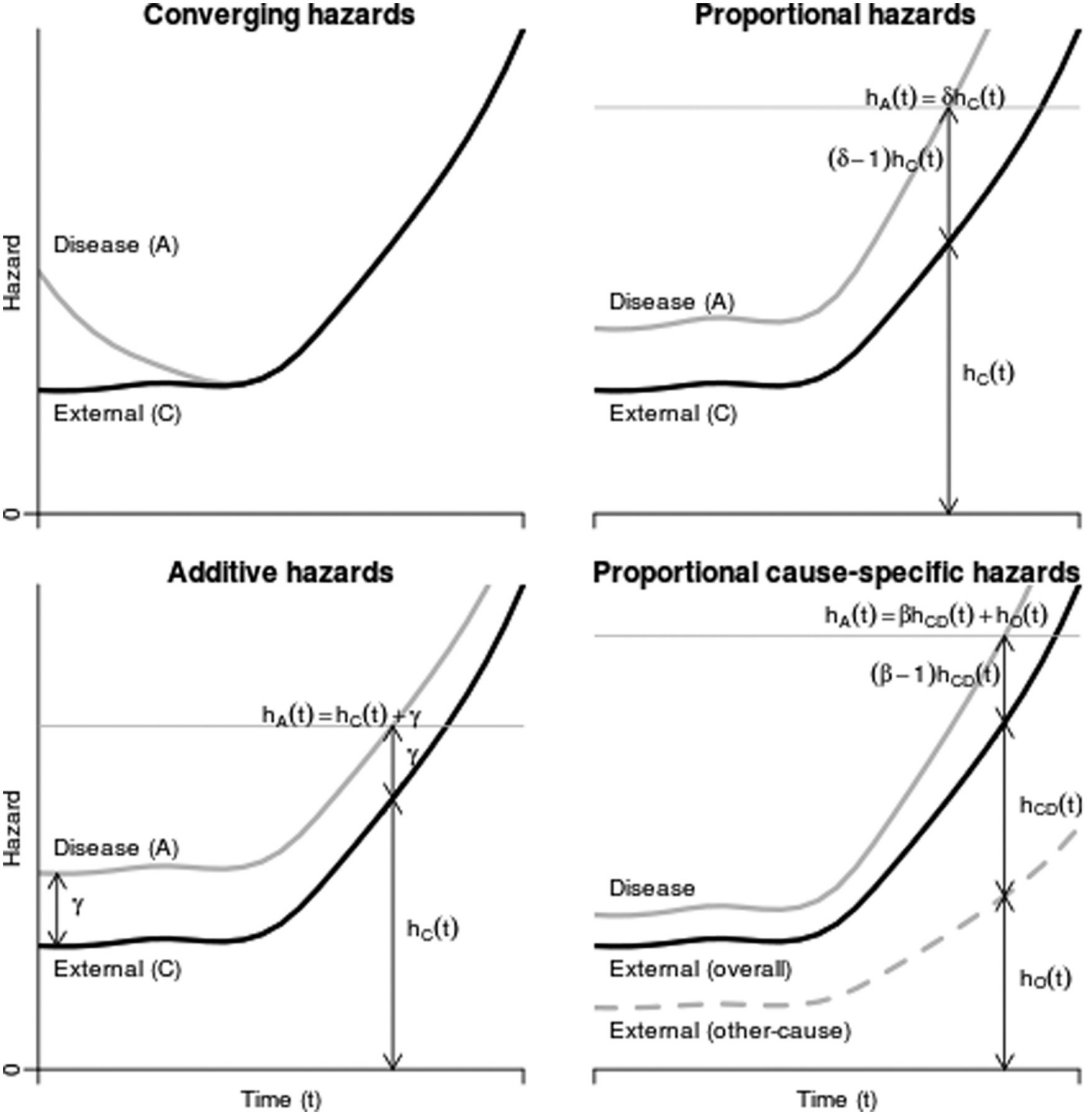
Example hazards for disease and external populations as functions of time, under 4 different assumptions about how the disease population hazards relate to the external population hazards.

In other cases, the disease population may have a higher initial mortality than does the general population, but this decreases until at some time (after $t=t_{c}$, say) its death rate converges to the mortality of that of the general population^20–29^ (Figure 3, top left).

$$h_{A}(t)=h_{B}(t)=h_{C}(t)\;\mathrm{for all t}>t_{c}.$$

If $t_{c}\leq t_{1}$, where $t_{1}$is the follow-up time of the RCT, survival for $t\leq t_{c}$ and $t>t_{c}$ can be taken directly from the trial data and the life-table data, respectively. Otherwise, if $t_{c}>t_{1}$, short-term extrapolations from parametric models fitted to the individual-level data from the RCT might be used to estimate the survival probability between $t_{1}$ and $t_{c}$.^25,29,30^ If the hazard is decreasing in the short term, extrapolating directly from a parametric model might then lead to hazards that are lower than those of the age/sex-matched general population, which is assumed to be implausible; therefore, using the life-table data is more appropriate. $t_{c}$ is sometimes interpreted as a “cure” time, so that all patients who survive this long are assumed to be “cured” and to have mortality equivalent to that of the general population. Messori and Trippoli^27^ also suggested that a compromise between “cured” population survival and “uncured” extrapolated survival might sometimes be appropriate—see the models originating from Boag,^31^ discussed later in this article, for examples.

### Disease and External Populations Have Different Mortality in the Short and Long Term

If the mortality of patients with the disease is different from that of the population represented by the external data at all times $t<t_{\max}$, then extrapolation might be achieved by adjusting the external evidence to make it more representative of the target population. This requires an assumption that mortality is systematically different between the populations in the long term, in a way that can be determined from the short-term data or informal beliefs. For example, there may be proportional or additive hazards for all-cause or cause-specific mortality between the disease and external populations. These assumptions are discussed in detail later.

## Difference in Mortality Between the Treatment and Control Populations

A similar decision should be made about the difference in mortality between the intervention and control groups (B and A, respectively). If the intervention is not expected to affect mortality (e.g., if it affects only quality of life), then $S_{B}(t)$ can be assumed to equal $S_{A}(t)$ for all times. If the relative intervention effect is expected to diminish to null soon after the end $t_{1}$ of the trial data, then $h_{B}(t)$ can be assumed to equal $h_{A}(t)$ in the long term, and it is sufficient to estimate $h_{A}(t)$.

$S_{B}(t)$ could then be estimated by combining a published relative treatment effect from trials,^32,33^ with the extrapolated $S_{A}(t)$. The assumptions required to do this are analogous to those required to extrapolate differences between the disease and external populations; typically, the hazard ratio between treatment groups for all-cause or cause-specific mortality might be assumed to be constant in perpetuity. Or, if individual data are available for the intervention as well as for the control arm of the trial, $S_{B}(t)$ could be produced independently of $S_{A}(t)$ by using external data and a similar method to that used to estimate $S_{A}(t)$. Even without external data, $S_{B}(t)$ and $S_{A}(t)$ are commonly estimated independently, by parametric extrapolation.^2^ This still assumes implicitly that the short-term differences between the treatment groups are representative of the long term. Bagust and Beale^30^ discuss how knowledge of the treatment’s mechanism of action might be used to guide long-term estimation; for example, the effects of a drug might take some time to achieve after starting treatment and dissipate gradually when treatment stops.

The assumption about how the relative treatment effect is likely to change as t increases from $t_{1}$, the end of trial follow-up, to the time horizon for the decision model is likely to be an important driver of which intervention is preferred.^34^ It is therefore important to consider uncertainty about this assumption. The fundamental problem is that information about this effect is available only in the trial data, not in the long-term data C. NICE^1^ recommends that 3 alternative scenarios be considered, corresponding to pessimistic, optimistic, and compromise assumptions about the long-term effect of a treatment that is effective in the short term. For example, expressing the effect as a hazard ratio $h_{B}(t)/h_{A}(t)$, the effect for $t>t_{1}$ could be

- null, so that $h_{B}(t)/h_{A}(t)=1$ for $t>t_{1}$;
- the same as in the short term, thus $h_{B}(t)/h_{A}(t)=\exp(\beta )$, assumed constant for all t; or
- diminishing in the long term, thus $h_{B}(t)/h_{A}(t)$ is increasing from exp(β) to 1.

Beyond informal sensitivity analysis, we did not find any literature where external information, such as elicited beliefs or the effects of related treatments with longer follow-up, was used formally to quantify future changes in expected treatment effects on survival.

## Adjusting External Data to Represent the Population of Interest

If patients with the disease (under either intervention) and the external population have different long-term mortality, then one of the following assumptions might be used to estimate $S_{A}(t)$ by adjusting the long-term external data, and similar methods might be used to estimate $S_{B}(t)$.

### Proportional Hazards for All-Cause Mortality between the Disease and External Populations

Several authors^35–37^ obtained cause-specific mortalities $h_{A}(t)$ by multiplying those estimated from life-tables $h_{C}(t)$ by a constant hazard ratio obtained from literature or literature combined with expert belief.^38^ These studies assumed proportional hazards; that is, the hazard ratio between the disease-specific and general populations is constant over time (Figure 3, top right).

$$h_{A}(t)=\delta h_{c}(t),\mathrm{equivalently}\;S_{A}(t)=S_{C}(t{)}^{\delta }.$$

This is sometimes implemented approximately by assuming the probabilities of death over a short period of time (e.g., the cycle length of a state-transition model) are proportional, instead of the hazards (the instantaneous rates of death, which are not probabilities^39^). Instead of taking the hazard ratio from the literature, Demiris and Sharples^40^ estimated it using a joint statistical model for the disease-specific and external data.

### Proportional Cause-Specific Mortality

The proportional hazards assumption can be convenient since comparisons of mortality between groups are often published as hazard ratios. However, all-cause mortality may not be proportional. For example, consider the causes of death that contribute to overall mortality. Let $h_{A}(t)=h_{\mathrm{AD}}(t)+h_{\mathrm{AO}}(t)$, where $h_{\mathrm{AD}}(t)$ is the hazard for disease-related mortality, and $h_{\mathrm{AO}}(t)$ is the hazard for mortality from all other causes in population A. Similar notation is used for populations B and C. Mortality from causes unrelated to the disease of interest can typically be assumed to be the same between patients with the disease and the external population, so that

$$h_{\mathrm{AO}}(t)=h_{\mathrm{BO}}(t)=h_{\mathrm{CO}}(t)=h_{O}(t).$$

Mortality for disease-related causes is typically higher. Suppose the hazards for disease-related mortality are proportional, so that $h_{A}(t)=\gamma h_{\mathrm{CD}}(t)+h_{O}(t)$ (Figure 3, bottom right). This is equivalent only to an all-cause proportional hazards model $h_{A}(t)=\delta h_{C}(t)=\delta (h_{\mathrm{CD}}(t)+h_{O}(t))$ if $h_{\mathrm{CD}}(t)/h_{O}(t)$ is independent of time. In other words, assuming proportional all-cause hazards would be valid only if disease-related mortality were a constant proportion of the overall mortality in the external population as time elapses. Benaglia and others^41^ estimated the likely extent of bias in various situations when this assumption is wrongly applied.

To implement a proportional cause-specific hazards model, estimates of $h_{\mathrm{CD}}(t)$and $h_{O}(t)$ can often be obtained from cause-specific population mortality rates published by national agencies. As with the all-cause hazard ratio, the cause-specific hazard ratio γ for disease populations relative to the external population might be obtained from the literature or estimated from short-term comparisons between internal and external data.^42–44^ The cause-specific hazard for the intervention group $h_{\mathrm{BD}}(t)$can be estimated similarly by multiplying $h_{\mathrm{AD}}(t)$ by a published constant treatment-specific hazard ratio, representing the effect of the intervention on cause-specific mortality. This supposes, however, that the causes of death targeted by the intervention are the same as the causes that distinguish the disease population from the general population, which may need to be investigated.^41^

In Benaglia and others,^41^ cause-specific death rates were published in the population life-tables; thus, $h_{\mathrm{CD}}(t)$and $h_{O}(t)$could be obtained easily. However, they were not published in the disease-specific individual-level survival data A. To overcome this and estimate γ, since the overall hazard for the disease population is defined as $h_{A}(t)=h_{\mathrm{AD}}(t)+h_{O}(t)$, a poly-hazard model^45^ could be applied, which decomposes the hazard for all-cause mortality as the sum of cause-specific hazards. Specifically, a poly-Weibull model was used for the internal data A, where the cause-specific hazards are both Weibull, and Weibull models were simultaneously applied to the external data. The common other-cause hazard assumption and proportional cause-specific hazard assumption then enabled the parameters of all hazard functions to be estimated through a joint model for populations A and C. This model implicitly assumes that the disease has no effect on hazards that have not been defined as disease-related in the external data, which cannot be tested unless deaths occurring in the internal trial patients also have the cause of death recorded.

A related method, originating from Boag,^31^ assumes a certain proportion of patients are cured and estimates a parametric survival function for the noncured patients. The cure fraction and the parameters of the noncured survival function are estimated jointly from individual data on survival and disease status. Hisashige and others^26^ and Maetani and others^46^ used this approach to obtain a disease-related survival curve $S_{\mathrm{AD}}(t)$ for the patients of interest, assuming that the noncured and cured survivor functions correspond to disease-related and disease-unrelated survival, respectively. A disease-unrelated survivor function $S_{\mathrm{CO}}(t)$ is obtained from age- and sex-matched life-table data. The overall extrapolated survivor function is then calculated as the product of the disease-related and unrelated survival, assuming equivalency to the above assumption of proportional cause-specific and identical other-cause hazards:

$$S_{A}(t)=S_{\mathrm{AD}}(t)S_{\mathrm{AO}}(t),\;S_{\mathrm{AO}}(t)=S_{\mathrm{CO}}(t).$$

### Additive Hazards for All-Cause Mortality between the Disease and External Populations

Instead of a constant risk ratio between internal and external data sources, some authors^47–49^ have assumed that the disease-specific population had a constant additive excess hazard compared to the general population (Figure 3, bottom left).

$$h_{A}(t)=h_{C}(t)+\alpha .$$

Under this assumption, it can be shown^47^ that $\mathrm{logit}(S_{A}(t)/S_{C}(t))$ converges to a linear function as *t* increases. Thus, the slope of a linear regression fitted to the latter part of observed data on $\mathrm{logit}(S_{A}(t)/S_{C}(t))$for $t<t_{1}$ gives an estimate of −α. Extrapolations of $S_{A}(t)$ for $t>t_{1}$ can then be calculated given the estimate of α. Demiris and Sharples^40^ also investigated additive hazard models within a Bayesian framework. An advantage of additive hazards is that cause-specific modeling is less important. If disease-related hazards are additive, so that $h_{\mathrm{AD}}(t)=h_{\mathrm{CD}}(t)+\alpha$ and then $h_{A}(t)=h_{\mathrm{CD}}(t)+\alpha +h_{O}(t)=h_{C}(t)+\alpha$, so the additive all-cause hazard model also holds, and the cause-specific risk difference α is equal to the all-cause risk difference $h_{A}(t)-h_{C}(t)$. The risk difference (or excess risk) is straightforward to interpret, and under the additive hazard model, it is independent of time. A proportional hazards model, however, is multiplicative, so that the excess risk depends on the baseline risk. Informally, the disease has a greater effect on people (such as older people) who are already at a higher risk of death, which is typical for a chronic disease.

The short-term fit of either the proportional or additive hazards assumption can be checked from the data by diagnostic plots^2,30^ or by embedding in a model that contains both as special cases, as discussed by Breslow and Day.^50^ The assumptions required to apply either in the long term, however, are untestable from data.

### Other Models for Parameterizing Mortality Differences between Populations

Other ways of parameterizing difference in survival between groups include accelerated failure time models, in which $S_{A}(t)=S_{C}(\delta t)$, so that the expected survival time in group C is δ times the expected survival time in group A, although we are unaware of these having been used in the context of survival extrapolation with external data. Nonproportional, nonadditive hazard models might also be used where the hazard ratio or excess hazard is a predictably varying function of time. For example, Andersson and others^51^ extrapolated survival of cancer patients by combining cancer cohort and life-table data and modeling the log cumulative excess hazard for cancer patients as a cubic spline function of log time,^52^ assuming a linear trend in the long term.

## Survival Model Choice When Combining Internal and External Data

To complete the estimation and to characterize the long-term differences between the disease and external population survival $S_{A}(t)$ and $S_{C}(t)$ as well as between the treatment and control survival $S_{B}(t)$ and $S_{A}(t)$, the form of each survival function needs to be specified.

Without external data, extrapolation of $S_{A}(t)$ and/or $S_{B}(t)$ is typically^2^ based on a parametric functional form for each survival curve. With external data, a parametric function could be specified for $S_{C}(t)$ and fitted to the external data and assumptions such as proportional hazards used to derive $S_{A}(t)$ and $S_{B}(t)$. To convert annual probabilities of death published in life-tables to individual-level survival times, which allows a survival model to be fitted, several authors^40,41,47,48^ have used simulation.

Alternatively, survival extrapolation can be performed semiparametrically with external data if these are available up to $t=t_{\max}$ and if a systematic difference between the external and internal populations can be assumed, such as proportional or additive hazards.^40,47,48^ This has the advantage of avoiding the risk of misspecifying the baseline survival function. Fang and others^47^ used semiparametric models, which gave plausible estimates where even a 3-parameter generalized gamma model did not. A hybrid approach is also possible, using nonparametric estimates up to some $t^{*}<t_{1}$ and parametric assumptions to extrapolate,^30^ although the results can be sensitive to the arbitrary choice of $t^{*}$.^53^

However, if the parametric form fits well, then fully parametric models can lead to greater precision in estimates.^54^ The advantages of parametric and semiparametric models are combined in a class of flexible parametric models based on modeling the log hazard as a spline, or piecewise cubic, function of log time,^52,55^ which can adapt to represent survival arbitrarily well. Since these models are fully parametric, they enable extrapolation beyond the times observed in the data.^56^ The spline function is defined to be smooth, and given a particular number of pieces, results have been shown to be not sensitive to the choice of where to subdivide the log time axis.^55^ Therefore, we would expect extrapolations from this model to be more robust than those from the “hybrid” approach mentioned above. Guyot and others^56^ used these models, implemented in the BUGS software,^57^ for survival extrapolation using a combination of trial and long-term external data. They can also be fitted to single survival data sets using Stata^58^ and R.^59^ Also, unlike the Cox model, they permit nonproportional hazards to be modeled^52^ and extrapolated if necessary.^51^

The choice between alternative parametric models for extrapolation is conventionally based on fit to the short-term data A, B.^2^ However, as recommended, for example, in the NICE guidelines,^1^ long-term plausibility should be considered based on external information such as knowledge of the disease, treatment and trial protocol,^30^ or related long-term survival data. External information could simply be used to inform the choice of model for extrapolation or to inform particular parameters of a chosen model. A plausible distribution might be chosen to represent how the hazard of death is expected to change over time. For example, the exponential distribution corresponds to a constant hazard, which is generally unrealistic in the long term as the hazard will increase as people get older. Therefore, even though data might suggest a constant hazard over the duration of the RCT, distributions that allow changes in hazards over time are likely to be more appropriate. Bagust and Beale^30^ also discuss how the apparent better fit of some parametric models may be an artifact of between-patient heterogeneity; for example, a Weibull distribution with shape less than 1 could be explained by a mixture of 2 subpopulations with different constant hazards.

Once the most appropriate model family has been chosen, its parameters can be estimated; this might be done using a combination of disease-specific data A and external evidence C. For example, Nelson and others^60^ used a 2-parameter Gompertz model, which has an exponentially increasing hazard, to extrapolate survival beyond the follow-up of an RCT. The parameter governing the baseline hazard was estimated using disease-specific data, and the hazard “acceleration” parameter was estimated from national population life-tables including older people.

When long-term data are not available or sparse, expert belief about long-term survival might be elicited to either choose the parametric form or estimate particular parameters, as we discuss later.

## Explaining Population Differences Through Observed Covariates

Under models such as the proportional or additive hazards specifications described above, the long-term difference between the populations underlying the trial and external data is characterized by a parameter such as the all-cause or cause-specific hazard ratio δ or γ or risk difference α. This is sufficient to estimate long-term survival of the trial population if the model assumptions hold. However, we may also want to explain this difference in terms of the characteristics of the people represented, for example, to estimate survival for subgroups of the population with certain characteristics. This is possible if relevant covariates are recorded in each source of evidence. Nelson and others,^60^ for example, used a proportional hazards model in which the log hazard ratio for all-cause mortality is a linear function of the covariates that distinguish the data sets. The covariate effects were estimated using a semiparametric model fitted to the long-term external data, to obtain an expression for survival S(t,x,β) as a function of covariate values x and covariate effects β. The survival for group A, $S_{A}(t)$, was estimated for all t by averaging S(t,x,β) over all covariate values x observed in the data A. This approach assumes that the form of the relationship with covariates is the same between populations A and C, which may not be true. For example, the relationship of the log hazard of death with age may be linear among younger people but nonlinear among older people.

It is common to assume that the increase in the hazard of death as a person gets older is fully explained by his or her increasing age. Thus, survival extrapolations often rely principally on modeling how the hazard increases with age. Population-based data commonly cover a wide range of ages and calendar periods. To exploit this diversity, Nelson and others^60^ fitted joint models to a combination of RCT and cohort data in an age metric, where the t in $S_{A}(t)$ and $S_{C}(t)$ represents age rather than time since diagnosis or randomization to treatment. This assumes that hazards change through time only with increasing age, although the shape of this dependence was modeled nonparametrically, with no further distributional assumptions.

Without long-term follow-up data, age effects on mortality could be estimated from shorter-term data on individuals with widely varying ages at baseline. Speight and others^13^ estimated long-term cancer survival using registry data in this way. The (within-person) increase in the risk of death as a person gets older was assumed to equal the risk ratio between people with different baseline ages.

## Representing Uncertainty and Parameter Estimation

It is important to characterize uncertainty in all model inputs and “structural” model choices^3^ in order to determine the uncertainty surrounding the treatment decision and assess the value of further research. In the presence of substantial decision uncertainty, the treatment might be recommended for use only in research or with otherwise limited coverage.^61^ If parameters used to extrapolate survival are estimated from data, the uncertainty inherent in estimating them can be handled by probabilistic methods. For example, in Fang and others,^47^ uncertainty about the estimation of the hazard increment β was propagated through the model to the estimated survival curve by bootstrapping. Alternatively, beliefs about β could be represented by a probability distribution in a standard probabilistic sensitivity analysis. Uncertainty about the choice of parametric model can be represented by choosing a sufficiently flexible model form, such as a spline-based or generalized gamma distribution,^56^ and, if the level of flexibility required is uncertain and different plausible models give different results, using model averaging.^62^

Bayesian methods are particularly suited to combining evidence from different sources in a model.^63^ The process involves defining a joint model with shared parameters representing the aspects that the different sources of data have in common (e.g., mortality for causes other than the disease of interest) and different parameters for the parts where they are expected to differ (e.g., cause-specific mortality). The posterior distributions of model outputs (such as incremental expected survival) are estimated simultaneously conditional on all data, and the uncertainty about the model inputs is propagated to the outputs. This approach has been used for combining data in the context of survival extrapolation,^40,41,56,64^ as well as in many other decision modeling contexts.^65,66^ External aggregate data or expert beliefs and associated uncertainty can be included as prior distributions, for example, published hazard ratios obtained from meta-analysis.^41^

A potentially more important uncertainty may arise in how the differences between the external and internal data are modeled—in other words, whether assumptions, such as those set out in this article, are valid in the long term. This is more problematic to identify from data; therefore, elicited beliefs might be used instead.

## Using Elicited Beliefs in Survival Extrapolation

Expert elicitation has been used to estimate uncertain quantities in health economic models,^67,68^ although we are unaware of this approach having been used in survival extrapolation. Here, we discuss the potential and challenges.

For example, beliefs about long-term survival might be elicited directly. Suppose that expert belief suggested that the 5-y survival probability, S(5|λ), (assuming $t_{1}<\;5$ *)* was most likely to be around 0.2 but could be as high as 0.3 or as low as 0.1. Assuming an exponential survival model, this belief about S(5|λ)=exp(−5λ) could be translated to a prior distribution for the rate λ=−log(S(5|λ))/5. Bayesian inference could then be used to combine this prior for long-term survival with the survival data for $t<t_{1}$. More complex and realistic parametric models would be more challenging. For example, in a Weibull model, eliciting expected survival $S(t|\alpha ,\lambda )=\exp(-\lambda t^{\alpha })$ could provide a distribution for $\lambda t^{\alpha }$, but extra assumptions would be needed to obtain separate priors for λ and α. To our knowledge, there has been no investigation of this. Survival estimates would need to be elicited at multiple time points to provide information about multiple parameters or to suggest an appropriate distributional form. Quantities are most easily elicited if expressed on an interpretable scale.^69^ Here, that could be the expected number out of 100 patients who will survive 5 y and 10 y, but it may be difficult to convert such information to priors for parameters. Expressing the elicited information as an artificial extra data set,^70^ then using standard methods to analyze the original data augmented with the additional data, may be a useful technique to investigate.

If some of the assumptions used to extrapolate are uncertain, then sensitivity analysis should be performed. The most basic form of sensitivity analysis is to present results under alternative scenarios and assumptions; however, scenario analyses can be difficult to interpret. Instead, the model might be extended by adding extra parameters representing these uncertain features, with prior distributions elicited from experts, then observing how the results are affected.^71^ For example, to assess the assumption of a constant hazard ratio between treatment groups, the treatment effect in the extrapolated period could be represented by a parametrically decreasing function of time, and plausible values for the parameter(s) could be elicited. This allows the associated decision uncertainty to be formally quantified and “value of information” methods used to determine whether it is worth doing further research to assess the assumption.^72^ Even without elicited information, informal beliefs could be used to demonstrate, for example, that the decision about which treatment would be preferred is robust within a plausible range of assumptions about some parameter. This might involve showing that the cumulative incremental net benefit of the intervention of interest is unlikely to cross the decision threshold in the period of time being extrapolated over.^73^

More research and experience are needed on the accuracy (and cost) of different methods to elicit uncertain quantities, ways to combine beliefs of different experts, what quantities should be elicited in this context, how best to use elicited information in models, and how the results can be communicated to decision makers.

## Summary and Research Priorities

Survival extrapolation given short-term data is a challenging task, involving prediction of data that have not been observed. Data on a related long-term population can often be exploited, but the necessary assumptions about how the populations differ, and how short-term trends might continue into the long term, must be clearly expressed and examined for plausibility and consistency with external data. This article reviews typical assumptions that might be made. However, we may sometimes not be confident in making any of these assumptions—it may be unclear whether the external data are relevant or how to explain differences between the data sets. The information required to adjust the external population to represent the internal population may not be available, for example, a marker of disease severity. In those cases, careful sensitivity analysis and characterization of uncertainty will be important. Since long-term assumptions, such as proportional hazards, are untestable from data, they should be clearly explained and justified to decision makers. More experience is needed in situations where neither proportional nor additive hazards assumptions are appropriate to distinguish the external and disease populations, and similarly when the treatment effect or other key parameters are not constant or otherwise predictable in the long term. Important open questions concern how “soft” information, such as formally elicited beliefs or the analyst’s own assumed distribution for uncertain quantities, can be obtained and used in modeling. Finally, we assumed that the trial data are representative of the target population that will ultimately receive the treatments of interest. This is not always true given the selection criteria of trials, although is more plausible for the phase III, pragmatic trials that typically inform cost-effectiveness models. Various authors^7–9^ have suggested methods and conditions for using external evidence to adjust the treatment effect from a trial to obtain the effect in an overlapping but nonidentical population. The covariate adjustment methods we discussed may also be used to explain differences in baseline survival between populations, if the relevant covariates are recorded.

## Supplementary Material

Supplementary material
