# Supplementary material for: Extrapolating Survival from Randomized Trials Using External Data: A Review of Methods
Source: Med Decis Making. 2016 Jul 10;37(4):377–90. doi: 10.1177/0272989X16639900 (PMC5424081; doi:10.1177/0272989X16639900)
Supplement: Supplementary material [file DS_10.1177_0272989X16639900.pdf]

APPENDIX: REVIEW METHODS

This work was motivated by a wider review of the currently-used methods used to extrapolate data from RCTs for use in health economic evaluations. This review took place in 2011, and used the methods described below to search three categories of literature. More recent literature was identified informally from our experience and by searching citations of key methodological papers – we do not expect a more systematic update of the review to reveal substantively new methods.

Literature A: NIHR Health Technology Assessment reports

We carried out a comprehensive review of health technology assessments performed under the National Institute for Health Research (NIHR) Health Technology Assessment (HTA) programme in the UK, both those commissioned by NICE and other reports. We extracted the full reports of the 214 HTA studies published between January 2004 and October 2010. The starting date was chosen to coincide with the first publication by NICE of their Guide to the Methods for Health Technology Appraisal. Each study report was reviewed and a decision made by one reviewer about whether the report involved the extrapolation of data from RCTs. At the third stage, those reports identified as relevant were categorised according to the type of data, measure, and variable used. Based on this information each report was reviewed to identify whether individual-level time-to-event data from RCTs were being extrapolated, and whether this involved the use of data external to the RCTs.
The decision at stage two about whether a report was relevant was made primarily by one author initially. These initial decisions were reappraised by at least one other author over the course of the review, as the authors developed a deeper understanding of what constitutes extrapolation as applied to RCTs in this context. A strictly formulaic approach was not adopted in order to benefit from this improvement in understanding of the methodological area other time. This meant the selection of HTA reports considered relevant was subject to a number of revisions over time.

Literature B: Health economics and medical statistics journal articles

Pearl-growing methods (10) were used to identify methods in the wider health economic literature. We firstly identified 29 key papers on extrapolation methods in the context of clinical trials and HTAs ("pearl" papers), using our own experience, expert external contacts and the results of an early scoping search. From each "pearl" paper, we reviewed reference lists and did a citation search to identify further papers containing substantial discussion on extrapolation issues. The pearl growing search process was iterative, with particularly pertinent papers being classified as 'new pearls' as the search progressed. The process was repeated for 3 iterations. 483 papers were

identified by the 1st iteration, 180 by the 2nd iteration and 59 by the final iteration. We also selected 5 key journals: Statistics in Medicine, Health Economics, Medical Decision Making, Value in Health and Pharmacoeconomics, and searched each for articles with a substantial discussion of extrapolation methods back to the year 2000. 310 papers ("key journal" papers) were found in searching these 5 key journals, and their abstracts were retrieved. Papers with no extrapolation content, or using methods already encountered in Literature A, were excluded. The full text of the 62 remaining papers was retrieved.

Literature C: Broader statistical literature

A more informal process was adopted to identify literature about methods which have not been used, but may potentially be used, for extrapolation in health technology appraisals. This involved a focussed search of Web of Science to identify existing classifications or taxonomies of extrapolation methods. As a comprehensive retrieval of potentially relevant papers was not the objective of the search, a selective list of terms was agreed and searched in the titles of indexed papers. These included variants of the terms 'extrapolation', 'predict' and 'forecast' combined with 'taxonomy' or 'classification'. Retrieved references were browsed and 63 potentially relevant references were selected and downloaded for further consideration.

24 HTA reports and 14 academic papers (all from Literature B) were finally identified containing methods for extrapolating survival from RCTs using external data. The methods used by each of these papers are broadly categorised in Table 1, corresponding to categories of assumptions described in our paper about the relationship between the baseline hazard of the external population and the patients of interest. Simpler assumptions are generally more common in applied work (HTA reports) compared to academic work. This may either be because those assumptions are reasonable more often than not, or because more complex methods were too technically demanding – we have not assessed whether the method used in each study was appropriate for that study.

	HTA reports	Academic literature	Total
Disease and external populations have the same baseline hazard at all times	11	0	**11**
Disease and external populations have the same baseline hazard after some time	5	4	**9**
External data adjusted parametrically to represent the baseline hazard of the population of interest for:			
All-cause mortality	5	7	**12**
Cause-specific mortality	3	3	**6**
**Total**	**24**	**14**	**38**

Table 1: Frequency of methods for survival extrapolation using external data, by type of literature
